# Baroreflex gain and time of pressure decay at different body temperatures in the tegu lizard, *Salvator merianae*

**DOI:** 10.1371/journal.pone.0242346

**Published:** 2020-11-23

**Authors:** Renato Filogonio, Karina F. Orsolini, Gustavo M. Oda, Hans Malte, Cléo A. C. Leite

**Affiliations:** 1 Department of Physiological Sciences, Federal University of São Carlos, São Carlos, São Paulo, Brazil; 2 Section for Zoophysiology, Department of Bioscience, Aarhus University, Aarhus C, Denmark; University of Georgia, UNITED STATES

## Abstract

Ectotherms may experience large body temperature (*T*_b_) variations. Higher *T*_b_ have been reported to increase baroreflex sensitivity in ectotherm tetrapods. At lower *T*_b_, pulse interval (PI) increases and diastolic pressure decays for longer, possibly resulting in lower end-diastolic pressures and mean arterial pressures (*P*_m_). Additionally, compensatory baroreflex-related heart rate modulation (*i*.*e*. the cardiac branch of the baroreflex response) is delayed due to increased PI. Thus, low *T*_b_ is potentially detrimental, leading to cardiovascular malfunctioning. This raises the question on how *P*_m_ is regulated in such an adverse condition. We investigated the baroreflex compensations that enables tegu lizards, *Salvator merianae*, to maintain blood pressure homeostasis in a wide *T*_b_ range. Lizards had their femoral artery cannulated and pressure signals recorded at 15°C, 25°C and 35°C. We used the sequence method to analyse the heart rate baroreflex-related corrections to spontaneous pressure fluctuations at each temperature. Vascular adjustments (*i*.*e*. the peripheral branch) were assessed by calculating the time constant for arterial pressure decay (τ)—resultant from the action of both vascular resistance and compliance—by fitting the diastolic pressure descent to the two-element *Windkessel* equation. We observed that at lower *T*_b_, lizards increased baroreflex gain at the operating point (*G*_op_) and τ, indicating that the diastolic pressure decays at a slower rate. *G*_op_ normalized to *P*_m_ and PI, as well as the ratio τ/PI, did not change, indicating that both baroreflex gain and rate of pressure decay are adjusted according to PI lengthening. Consequently, pressure parameters and the oscillatory power fraction (an index of wasted cardiac energy) were unaltered by *T*_b_, indicating that both *G*_op_ and τ modulation are crucial for cardiovascular homeostasis.

## Introduction

Temperature is possibly the most important abiotic factor affecting the physiology of ectotherms [1]. Increased body temperature (*T*_b_) is associated with higher heart rate (*f*_H_) and cardiac output [2–7], although mean arterial pressures (*P*_m_) are less affected [4,7–9]. In vertebrates, acute changes in arterial blood pressure are regulated by the baroreflex mechanism [10]. The cardiac branch of the baroreflex response is expressed as baroreflex gain (*i*.*e*. the heart rate variation per unit of pressure change [11]). The maximum baroreflex gain (*G*_50_) is temperature-sensitive in amphibians [7] and reptiles [12], and exhibits higher values at elevated *T*_b_.

At lower *T*_b_, the increased pulse interval (PI) resultant from lower *f*_H_ necessarily imply delayed and slower baroreflex-related *f*_H_ modulations to rapid arterial pressure variations. Additionally, the extended interval between heartbeats allows for elongated periods of diastolic pressure decay, potentiating the likelihood for hypotension. Under these circumstances, an inefficient response of the cardiac branch of the baroreflex mechanism could result in a hindered tissue perfusion. Notwithstanding, free-ranging amphibians and reptiles experience a broad range of *T*_b_ [1,13–22] without any apparent cardiovascular malfunction or homeostasis impairment. Therefore, other mechanisms must compensate for the loss of efficiency of the cardiac branch of the baroreflex at low *T*_b_.

**Fig 1:**
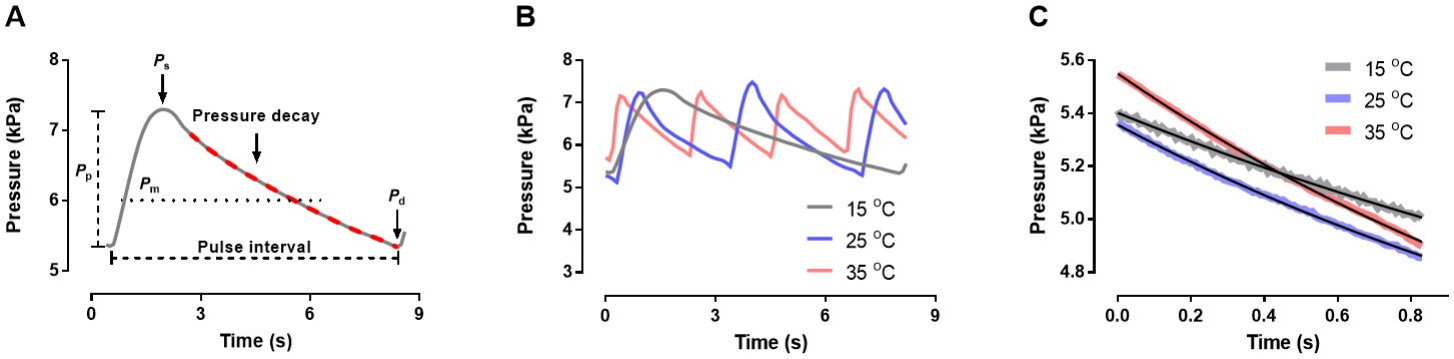
Representative original pressure traces recorded from tegu lizards, *Salvator merianae*. **A)** Pressure (in kPa) of a *S*. *merianae* recorded at 15°C (n = 1). The scheme indicates the peak systolic pressure (*P*_s_), end-diastolic pressure (*P*_d_), pulse pressure (*P*_p_), mean arterial pressure (*P*_m_; closed circle), diastolic pressure decay (red broken line), and pulse interval. **B)** Pressure (in kPa) recorded at 15°C (grey line), 25°C (blue line) and 35°C (red line) (n = 1). **C)** Example of estimated pressure decay using the *Windkessel* equation (black lines), based at the diastolic pressure recorded at 15°C (grey line), 25°C (blue line) and 35°C (red line) (n = 3).

We speculated that the vascular branch of the baroreflex could assist the cardiac branch in sustaining the cardiovascular homeostasis when *T*_b_ reduces. One way of assessing vascular regulation is by analyzing the rate of diastolic pressure decay (*i*.*e*. the time constant for arterial pressure decay during diastole: τ; Fig 1A), which is the result of the action of both vascular resistance and compliance when aortic valves close [23]. We expected that, since vascular resistance increases at colder *T*_b_ [4,8], diastolic pressure (*P*_d_) should exhibit a slower decline, thus avoiding hypotension. In addition, a slower pressure decay could minimize pressure oscillations around *P*_m_ at lower *T*_b_, thus reducing the relative “wasted” cardiac energy (*i*.*e*. the oscillatory power fraction–OPF [24]). Therefore, we postulated that the control of the vascular system by the peripheral branch of the baroreflex would compensate the loss of efficiency of the cardiac branch in maintaining pressure homeostasis by regulating τ according to PI lengthening. This could prevent both hypotension and larger oscillations of blood pressure when *T*_b_ decreases.

The present study was designed to investigate putative functional adjustments of both cardiac and peripheral branches of the baroreflex that allow effective pressure regulation in a range of *T*_b_ experienced by the tegu lizard, *Salvator merianae*. The study was conducted during the non-reproductive period of the species, when facultative endothermy is not manifested and *T*_b_ varies as a typical ectotherm lizard [25]. We used the sequence method to assess baroreflex gain at the operating point (*G*_op_; *i*.*e*. gain at the point of the baroreflex sigmoidal curve correspondent to the *P*_m_ operating point [26]) and baroreflex effectiveness index (BEI; *i*.*e*. the capacity of the baroreflex to overcome concomitant stimuli modulating *f*_H_ [27,28]) to investigate the responses of the cardiac branch. Since longer PI at lower *T*_b_ enables pressure to decay for longer times, it is possible that *G*_op_ is adjusted to protect against hypotensive episodes. We also investigated τ and the ratio τ/PI as an estimate of vascular regulation at different *T*_b_. We predicted that in order to preserve systemic functionality, τ and PI should variate accordingly as to maintain τ/PI and OPF relatively constant. Hence, baroreflex should trigger compensatory adjustments that involve both cardiac and vascular responses to *T*_b_ changes in *S*. *merianae*.

## Material and methods

### Animal acquisition and maintenance

Eleven juvenile tegu lizards (*Salvator merianae*, mean mass ± standard deviation: 622.7 ± 90.6 g) were donated by the Jacarezário (UNESP–Rio Claro, Brazil), and maintained at the facilities of the Laboratory of Experimental Biology (UFSCar–São Carlos, Brazil). Animals were kept in groups of four to five individuals at 1.2 × 0.8 × 0.8 m tanks with access to heating lamps, under a natural light regime ~12:12 h. Tegu lizards were fed on eggs and chicken liver and had access to *ad libitum* water supply. Feeding was interrupted for five days (equivalent to the postprandial duration after 10% of body mass ingestion in *S*. *merianae* maintained at 30°C [29]) prior to experimental procedures to avoid SDA effect on the cardiovascular parameters.

### Instrumentation

Before surgical procedures, lizards were sedated with elevated levels of CO_2_ until complete loss of righting reflexes [28,30–33]. Individuals underwent tracheal intubation and were mechanically ventilated with isoflurane (2–5%; 5 breaths × min^-1^; tidal volume of 30 ml × kg^-1^; SAR-830/P Ventilator) throughout the entire surgical procedure. A heating cushion set to 30°C was used to maintain a stable body temperature. Local anaesthetic (Lidocaine 2%, Pearson; 10 mg × kg^-1^) was injected in the left thigh before a 3 cm longitudinal incision was made. The femoral artery was occlusively cannulated with a P50 catheter filled with heparinized saline (50 IU × ml^-1^). Lizards were injected with antibiotic (Chemitril, 11mg × kg^-1^) and anti-inflammatory/analgesic (Flunixin 1.1mg × kg^-1^) just after the surgical procedure, and after every 48 hours for four days. All procedures were performed under sterile conditions. Animals were allowed to recover in a temperature-controlled chamber set to 35°C (which is within the species’ preferred body temperature range [25,34]) in a maintenance container (25 × 35 × 10 cm). Experimental protocols started five days after the instrumentation surgery, which corresponds to the recovery time of the resting pattern of autonomic modulation after instrumentation in *S*. *merianae* [30].

Before measurements, the catheter was connected to a Baxter Edward (model PX600, Irvine, CA, USA) disposable pressure transducer and signals were amplified with a single-channel preamplifier (Bridge Amp, ADInstruments) before being connected to a Power Lab^®^ data acquisition system (ADInstruments). Pressure transducers were daily calibrated against a static water column before measurements using LabChart^®^ software (LabChart v.7.0, ADInstruments).

Throughout the recovery period, the catheter was washed with sterile heparinised saline and signal quality was evaluated in order to check for any signal loss. During the protocol, lizards were exposed to one of the three experimental temperatures (35, 25 or 15°C) each day, in a decreasing order. Lizards were given 24 h to allow *T*_b_ stabilization at each set temperature before pressure recording. Tattersall et al. [25] reports that adult tegu lizards (~ 2000 g) require less than 10 h to cool down approximately 14°C inside their burrows. Therefore, the 24 h interval between measurements in the present study was sufficient for lizards to stabilize their *T*_b_ with the set environmental temperature. Accordingly, blood pressure traces were recorded for 2–3 h from autonomic recovered resting lizards at different *T*_b_ encompassing the temperature range most commonly experienced by *S*. *merianae* [25].

By the end of the protocol, lizards were anaesthetized and euthanized by injection of thiopenthal (Thiopentax, Cristália; 50 mg × kg^-1^), followed by an i.v. injection of a saturated K^+^ solution until the heart stopped beating. All procedures were performed in accordance with guidelines from the Brazilian National Council for the Control of Animal Experimentation (CONCEA), and approved by the Ethics Committee on Animal Use of the Federal University of São Carlos (CEUA/UFSCar n° 4663270916).

### Data analysis

Before analysis, pressure signals were filtered using a low pass (20Hz) digital filter. For each temperature tested, peak systolic and end-diastolic arterial pressures (*P*_s_ and *P*_d_, respectively), heart rate (*f*_H_) and pulse interval (PI) were obtained using the distance between consecutive diastolic pressures (Fig 1A). Mean arterial pressure (*P*_m_) was calculated as *P*_d_ + (*P*_s_−*P*_d_) / 3, whereas pulse pressure (*P*_p_) was the difference between *P*_s_ and *P*_d_ (Fig 1A) [35].

We utilized the sequence method to assesses the baroreflex at the operating point (*G*_op_) based on the average of the slopes from spontaneous baroreflex sequences (*i*.*e*. minimum of three cardiac cycles displaying sequential increases or decreases in *P*_s_ followed by concomitant modulation of PI [27,28,36]). Baroreflex gain obtained was then normalized for *P*_m_ and PI to allow for meaningful comparisons between temperatures [37,38]:

$$G_{\text{norm}}=G_{\text{op}}(P_{\text{m}}/\text{PI})$$ (1)

Baroreflex effectiveness index (BEI) was calculated as a ratio between the number of baroreflex sequences and the total number of ramps, which comprise both baroreflex and non-baroreflex sequences [27,28]:

$$\text{BEI}=\frac{\text{Number}\;\text{of}\;\text{baroreflex}\;\text{sequences}}{\text{Total}\;\text{number}\;\text{of}\;\text{ramps}}$$ (2)

These calculations were performed with CardioSeries software (v2.4, www.danielpenteado.com) utilizing a minimum of 300 cardiac cycles and delay 1 [28].

To assess the putative regulation of the vascular system to different *T*_b_, we calculated the time constant of arterial pressure decay during diastole (τ; Fig 1A). We fitted a representative portion of the second half of the diastolic pressure curve to a modified two-element *Windkessel* equation based on Westerhof et al. [23]:

$$P(t)=(P_{0}-A){\text{e}}^{-t/\tau }+A$$ (3)

Where *P*(*t*) is diastolic pressure at time *t*, *P*_0_ is end-systolic pressure, and *A* is the asymptote [23,39,40]. Values were fitted using GraphPad Prism v.7.00.

We calculated oscillatory power fraction (OPF) as determined by Saouti et al. [24]:

$$\text{OPF}=1-P_{\text{m}}/P_{\text{s}}$$ (4)

Data were analysed using one-way ANOVA for repeated measures using temperature as factor followed by Tukey *post hoc* test using SigmaPlot (v. 11). Normality was assessed with a Kolmogorov-Smirnov test. Statistical significance was assigned as P < 0.05. Data are presented as mean ± standard deviation.

## Results and discussion

Acknowledging that adult *S*. *merianae* produce internal heat during the reproductive season [25], we studied juveniles during the non-reproductive period to avoid this confounding factor. Nonetheless, it is worth noting that the preferred *T*_b_ of *S*. *merianae* does not depend on size or reproductive condition [34]. Heart rate exhibited the typical increase with *T*_b_ as observed in other reptiles [3,8,41,42], with the concomitant decrease in PI (Table 1; Fig 1B). In a recent investigation, O_2_ consumption of tegu lizards increased about 4-fold between 17°C and 37°C [43], and *f*_H_ followed a similar pattern in the present study. This indicates that cardiac output regulation supporting metabolic alteration triggered by temperature change is mainly governed by *f*_H_ modulation. The close relationship between *f*_H_ and metabolic rate has been experimentally evidenced for *S*. *merianae* [43]. Despite the magnitude of those alterations, none of the pressure parameters changed with *T*_b_ (Table 1). This agrees with results reported for the freshwater turtle *Trachemys scripta*, where *P*_m_ remains unchanged upon *T*_b_ variation [4].

**Table 1:** Temperature effects on the hemodynamic variables.

Variable	Body temperature			ANOVA	
	15°C	25°C	35°C	F_2,31_	P
*f*_H_ (bpm)	12.83±4.17^a^	22.48±9.49^b^	42.74±19.10^c^	37.611	<0.001
PI (s)	5.04±1.28 ^a^	3.14±1.32 ^b^	1.62±0.58 ^c^	34.974	<0.001
*P*_s_ (kPa)	6.24±1.47	7.08±1.27	7.24±1.73	1.646	0.219
*P*_d_ (kPa)	4.35±0.94	4.95±0.96	5.15±1.41	1.524	0.243
*P*_m_ (kPa)	4.98±1.09	5.66±1.03	5.84±1.50	1.591	0.230
*P*_p_ (kPa)	1.89±0.76	2.13±0.66	2.09±0.61	1.003	0.385
*G*_op_ (s × kPa^-1^)	5.46±2.60^a^	5.00±5.02^a^	1.52±0.66^b^	7.585	0.004
*G*_norm_ (unitless)	5.25±2.07	7.95±5.80	5.39±1.82	2.065	0.154
BEI (unitless)	0.42±0.13	0.31±0.14	0.37±0.09	2.988	0.074
τ (s)	5.87±2.56^a^	2.90±1.43^b^	1.92±1.01^b^	16.472	<0.001
τ/PI (unitless)	1.21±0.58	0.99±0.43	1.25±0.69	0.678	0.520
OPF (unitless)	0.20±0.06	0.20±0.05	0.19±0.05	0.158	0.855

*f*_H_ = heart rate; PI = pulse interval; *P*_s_ = systolic pressure; *P*_d_ = diastolic pressure; *P*_m_ = mean arterial pressure; *P*_p_ = pulse pressure; *G*_op_ = baroreflex gain at the operating point; BEI = baroreflex effectiveness index; τ = pressure decay time constant; τ/PI = ratio between pressure decay time constant and pulse interval; OPF = oscillatory power fraction. Different letters denote statistical differences according to temperature changes (one-way ANOVA for repeated measurements and Tukey test; P < 0.05). Data are presented as mean ± s.d. (n = 11).

**Fig 2:**
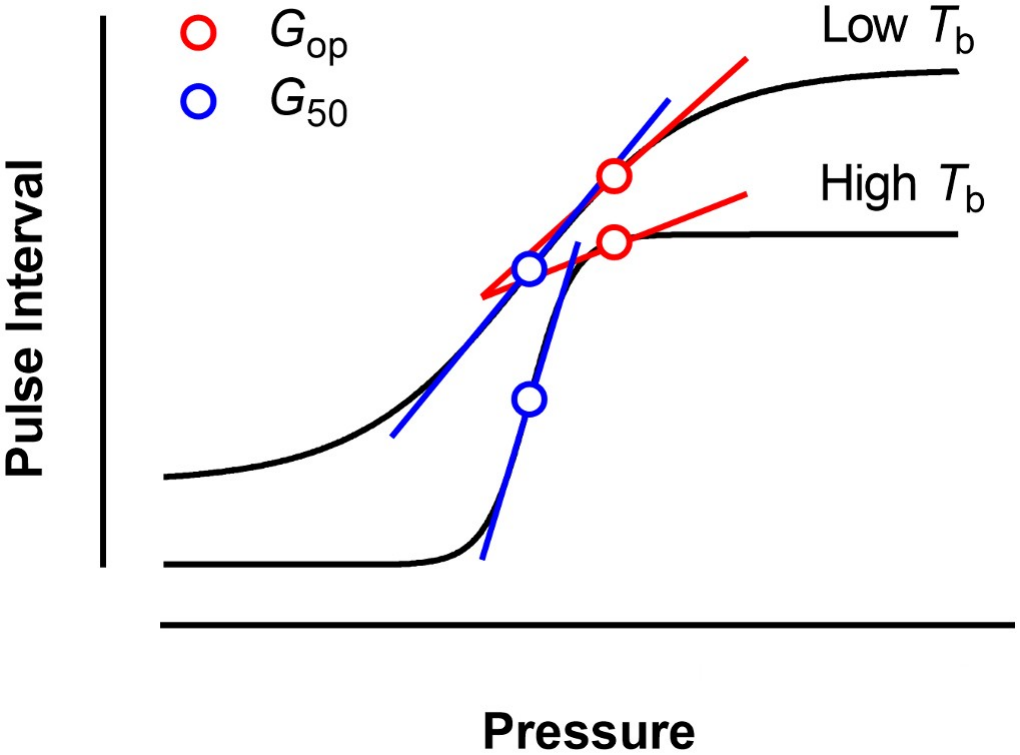
Schematic figure comparing baroreflex sensitivity assessed by two different methods. The sigmoidal baroreflex curves represent a theoretical response to body temperature (*T*_b_) changes. The sequence method estimates gain at the operating point (*G*_op_; red circle), whereas the pharmacological method estimates maximum gain (*G*_50_; blue circle). The slope at the specific point of the curves are in red for *G*_op_, and blue for *G*_50_. Note that, while the slope at *G*_op_ is less inclined at higher *T*_b_, slope at *G*_50_ is steeper.

*G*_op_ was the lowest when measured at 35°C (Table 1). This result is in stark contrast with those from other ectotherms, where baroreflex sensitivity was shown to increase with temperature [7,12]. This may be due to differences between the sequence method and the pharmacological method (*i*.*e*. the Oxford method) regarding the calculation of baroreflex gain. The sequence method used in the present study estimates baroreflex gain close to the *P*_m_ at the operating point, whereas the pharmacological method used in previous studies [7,12,38,44,45] calculates maximum gain [26]. The *P*_m_ at the operating point for *S*. *merianae* was estimated to be higher than *P*_m_ at the midpoint of the *f*_H_ baroreflex response range [44], which is used to calculate *G*_50_ [46]. Therefore, the two methods estimate gain at different regions of the baroreflex response curve (Fig 2). A steeper slope at the midpoint of the *f*_H_ baroreflex response range increases *G*_50_. We speculate that, when *T*_b_ increases, the slope of the baroreflex sigmoidal curve at the operating point decreases, whereas the slope at the midpoint of the *f*_H_ baroreflex response range increases at the same conditions (Fig 2). In this way, it is possible that increased *T*_b_ could induce reductions in *G*_op_ at the same time *G*_50_ increases.

Nonetheless, the higher *G*_op_ at 15°C and 25°C indicates the sensitivity for correction of arterial pressure perturbations are enhanced. Since longer PI leaves more time for pressure to decay, it is possible that this increased *G*_op_ helps *S*. *merianae* to better protect against hypotension. The unaltered normalized gain values (*G*_norm_; Table 1) indicate that the baroreflex sensitivity in the tegu lizard is actively optimized to work at the different *f*_H_ and blood pressure conditions imposed by different *T*_b_. This is further substantiated by the unaltered BEI over temperature changes (Table 1).

The two-element *Windkessel* model (Eq 3) fitted well to our dataset (R^2^ > 0.999; Fig 1C). Increased temperatures led to decreased τ (Table 1), probably as a result of reduced arterial resistance [4,8,47], indicating pressure during diastole falls faster at 25°C and 35°C. However, the ratio τ/PI was similar at all temperatures tested (Table 1), meaning that the time for pressure decay was proportional to pulse interval. A constant relationship between τ and PI was also observed for mammals where *f*_H_ decreases as an effect of scaling with body size, and was argued as the reason why *P*_p_ and *P*_d_ were unaltered [48]. Likewise, the constant *P*_p_ and *P*_d_ exhibited by *S*. *merianae* in the present study are probably the result of the proportional changes of τ related to PI. This conclusion was supported by the unvarying OPF at all temperatures (Table 1), indicating that the relative energy expended by the heart at each cardiac cycle remains constant (~18–20% energetic waste) throughout the temperature gradient experienced by resting *S*. *merianae* in our experiments.

The present study was the first to evaluate the efficiency of the orchestrated baroreflex response from both heart rate and vascular regulation to temperature variations in an ectotherm vertebrate. Here, we demonstrated that both responses are adjusted in concert to regulate the arterial blood pressure at different *T*_b_. For example, *G*_op_ was exacerbated when *T*_b_ dropped from 35°C to 25°C, possibly as a stronger response to hypotension since pressure decayed for longer and τ was similar between these two *T*_b_. On the other hand, τ increased when *T*_b_ reduced from 25°C to 15°C, while *G*_op_ remained unchanged. Those adjustments ensured similar *P*_m_ at all *T*_b_ tested, and prevented the amplification of pressure oscillations when PI increased, thus minimizing the cyclic waste of cardiac energy. Therefore, the present data underlines the fundamental role of the vascular regulation, in addition to the baroreflex-related heart rate response, in sustaining blood pressure homeostasis and cardiac efficiency of *S*. *merianae* at different *T*_b_.

## Supporting information

S1 Data (TXT)

S2 Data (CSV)

## Abbreviations

BEI: baroreflex effectiveness index
*f*_H_: heart rate
*G*_50_: maximum baroreflex gain
*G*_norm_: normalized baroreflex gain
*G*_op_: baroreflex gain at the operating point
OPF: oscillatory power fraction
*P*_d_: end-diastolic pressure
PI: pulse interval
*P*_m_: mean arterial pressure
*P*_p_: pulse pressure
*P*_s_: peak systolic pressure
*T*_b_: body temperature
τ: time constant for arterial pressure decay during diastole
